# Predicting the impact of climate change on the potential distribution of the invasive tomato pinworm *Phthorimaea absoluta* (Meyrick) (Lepidoptera: Gelechiidae)

**DOI:** 10.1038/s41598-023-43564-2

**Published:** 2023-09-30

**Authors:** Abdelmutalab G. A. Azrag, Francis Obala, Henri E. Z. Tonnang, Brian N. Hogg, Shepard Ndlela, Samira A. Mohamed

**Affiliations:** 1 International Centre of Insect Physiology and Ecology (icipe), P.O. Box 30772, Nairobi, 00100 Kenya; 2 Invasive Species and Pollinator Health Research Unit, USDA-ARS, Albany, CA 94710 USA

**Keywords:** Ecology, Zoology, Ecology, Environmental sciences

## Abstract

*Phthorimaea absoluta* (Meyrick) (= *Tuta absoluta*) (Lepidoptera: Gelechiidae), is the most damaging insect pest threatening the production of tomato and other solanaceous vegetables in many countries. In this study, we predicted the risk of establishment and number of generations for *P. absoluta* in the current and future climatic conditions under two Shared Socioeconomic Pathways (SSP2-4.5 and SSP5-8.5) of the years 2050 and 2070 using insect life cycle modelling (ILCYM) software. We used a temperature-dependent phenology model to project three risk indices viz., establishment risk index (ERI), generation index (GI), and activity index (AI) based on temperature data. The model projected large suitable areas for *P. absoluta* establishment in the Southern hemisphere under current and future climatic scenarios, compared to the Northern part. However, the risk of *P. absoluta* is expected to increase in Europe, USA, Southern Africa, and some parts of Asia in the future. Under current conditions, *P. absoluta* can complete between 6 and 16 generations per year in suitable areas. However, an increase in GI between 1 and 3 per year is projected for most parts of the world in the future, with an increase in AI between 1 and 4. Our results provide information on the risk of establishment of *P. absoluta* which could guide decision-makers to develop control strategies adapted for specific agro-ecological zones.

## Introduction

Globally, the introduction of invasive species in new environments outside their native range is increasing at unprecedented rates^1,2^. This could be further exacerbated by climate change^3^. Once invasive species arrive and establish themselves in a new environment, they can pose serious threats to crop production if they are not effectively managed. This was the case with the invasion by the South American tomato pinworm, *Phthorimaea absoluta* (Meyrick) (= *Tuta absoluta*) (Lepidoptera: Gelechiidae). Since its first report in Spain in 2006, *P. absoluta* has rapidly spread and become established in many countries across the Afro-Eurasian Supercontinent, causing devastating yield losses, especially for solanaceous vegetables^4,5^. Over seventeen years after its first detection, *P. absoluta* continues to expand its geographical range with the latest invasion reported in Togo^6^ in West Africa and China in Asia^7^, the largest global producer of tomatoes. The success of invasion and the subsequent establishment of *P. absoluta* is attributed to its innate dispersal ability, high reproductive rate, short life cycle^5^, wide thermal tolerance, and high phenotypic plasticity^8–10^.

Although tomato is the primary host of *P. absoluta*, the pest also attacks and completes its life cycle on other wild and cultivated plants belonging to the family Solanaceae such as the black nightshade, *Solanum nigrum* L., potato, *Solanum tuberosum* L. and eggplant, *Solanum melongena* L.^11^. The damage is caused by larval feeding on the leaves, flowers, stems, and fruits, causing tunnels that disrupt sap flow and photosynthesis. The infestation of *P. absoluta* on tomato fields is highly detrimental, and it can cause a yield loss of up to 100% if it is left uncontrolled^4^, leading to severe economic impacts, especially for small-scale farmers^12^. Although several attempts have been made to manage the pest using eco-friendly strategies^13–15^, the application of synthetic insecticides remains the main practice for controlling *P. absoluta*^16^. Therefore, a better understanding of the ecological traits and distribution of *P. absoluta* could help in developing effective control measures adapted to specific agroecological zones.

Over the last few decades, modelling the responses of insect pests to climatic factors, and predicting their geographical distribution, abundance, and risk of invasion have gained much interest^17–19^. Temperature is known to be the key abiotic factor that affects the distribution, abundance, and population dynamics of insects^18,20^. In the context of climate change, the increase in temperature from 1.5 to 5.8 °C by the end of the twenty-first century^21^ is likely to have either direct or indirect effects on the population of many insect pests^22^. The temperature increase could directly affect insects' life histories, physiology, and behavior^23^. The indirect effect is expected to be through the interactions between the pests and their host plants and natural enemies, which in turn might influence the distribution and population dynamics of insect pests^22^. Under favorable climatic conditions and in the absence of natural enemies, rapid multiplication of the pests occurs, and this might result in huge crop losses. In this regard, predicting the responses of insect pests to climate change has become more relevant for assessing the risk of invasion and developing ecologically sound management strategies.

Climate-based simulation models are important tools for forecasting pests’ risk in agricultural ecosystems under different climatic scenarios. Two types of pest simulation models, namely inductive and deductive are used to predict the distribution, abundance, and ecological niche of pests based on climatic data^20,24^. The inductive method relies on the environment of the localities in which the insect species is found. This method correlates the occurrence of the species to environmental data of each location to generate the probability of occurrence under different climatic scenarios^25,26^. On the other hand, the deductive method is based on the thermal requirements for pest development (thermal thresholds for development), which are obtained by fitting linear and nonlinear functions to insect development, survival, and fecundity^20^. In addition, the thermal requirements of the pest are incorporated with climatic data into a geographic information system (GIS) to simulate the impact of climate change on the protentional distribution of the pest in a given location^17,20^.

The potential distribution of *P. absoluta* under a warming climate was projected by four different CLIMEX models^4,27–29^ using information from literature, specifically on the pest occurrence data and its thermal requirements. Among these, two models projected the potential distribution of the pest worldwide^27,29^, while others focused the projection on a regional level. Although CLIMEX combines both inductive and deductive approaches to simulate pest distribution, the thermal requirements data for *P. absoluta* used in these studies to adjust models’ parameters were not validated under field conditions. More recently a study by Fand et al.^30^ predicts the invasion risk of *P. absoluta* in India using the ecological niche Maxent model under the current and future climatic scenarios. However, none of the above studies projected the future distribution of *P. absoluta* under different Shared Socioeconomic Pathways (SSPs) (i.e., SSP1-2.6, SSP2-4.5, SSP4-6.0, and SSP5-8.5). In addition, different modelling approaches for *P. absoluta* are required to enrich the existing literature for a better understanding of the distribution of the pest under changing climate. Therefore, this study aimed to predict the impact of climate change on the future distribution of *P. absoluta* under two SSPs (SSP2-4.5 and SSP5-8.5) for the years 2050 and 2070 using Insect Life Cycle Modeling (ILCYM) software^17,20^.

## Results

### The establishment risk index (ERI) of *Phthorimaea absoluta*

**Figure 1:**
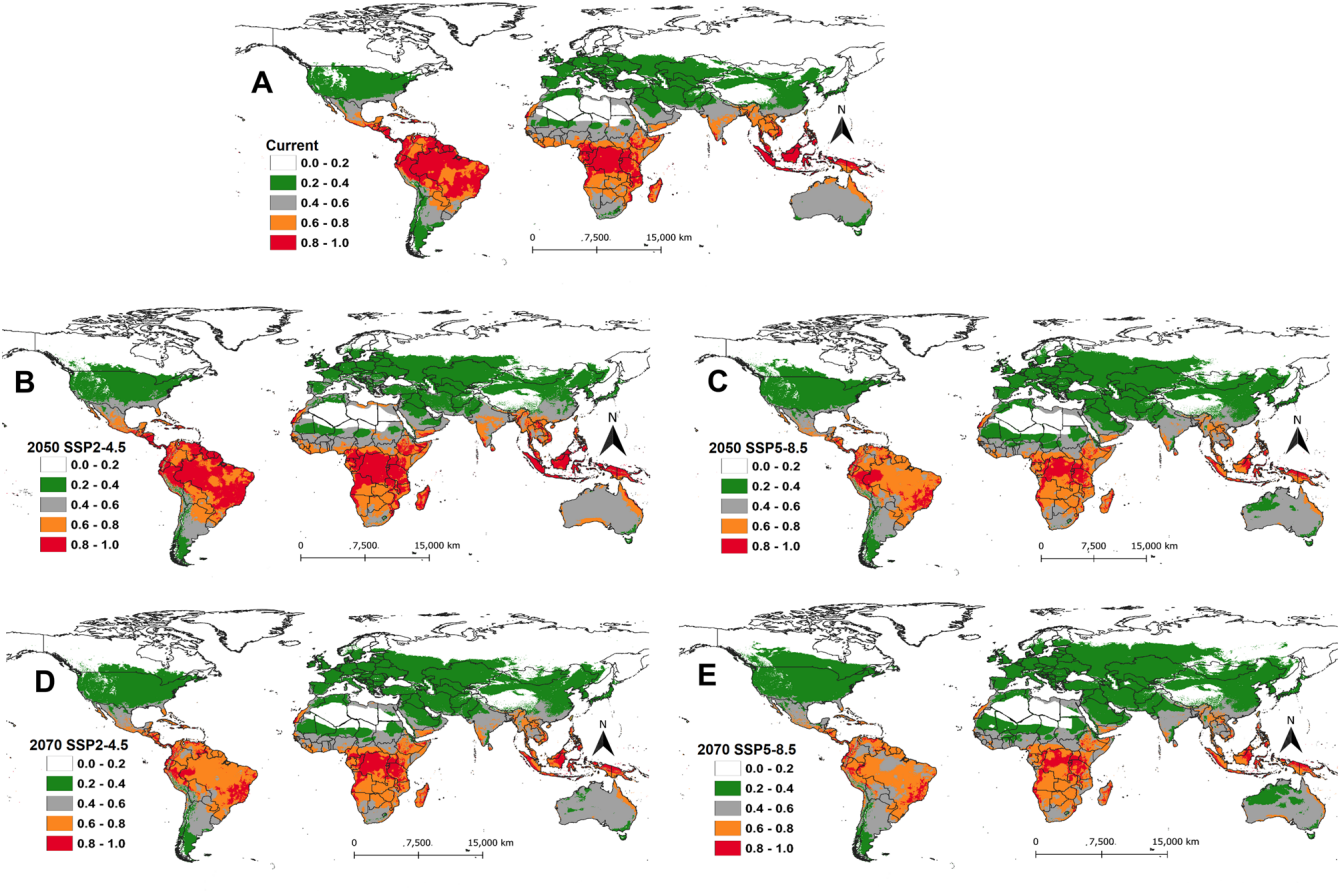
The establishment risk index (ERI) of *Phthorimaea absoluta* projected worldwide using temperature-based phenology model under (**A**) current climatic conditions, (**B**) future scenarios for 2050 (SSP2-4.5), **(C**) future scenarios for 2050 (SSP5-8.5), (**D**) future scenarios for 2070 (SSP2-4.5) and (**E**) future scenarios for 2070 (SSP5-8.5). The areas with ERI > 0.6 indicated that *Phthorimaea absoluta* could permanently establish.

We classified the establishment risk index (ERI) into five classes viz., unsuitable areas (ERI = 0.0–0.2), marginally suitable areas (ERI = 0.2–0.4), suitable areas (ERI = 0.4–0.6), highly suitable areas (ERI = 0.6–0.8) and optimal areas for survival (ERI ≤ 0.8). Overall, most parts of the Southern hemisphere of the world are projected to be highly suitable for *P. absoluta* establishment compared to Northern hemisphere under both current and future climatic scenarios (Fig. 1). Under the current climatic conditions, the pest could permanently establish (ERI ≤ 0.6) in most parts of sub-Saharan Africa except for the Sahara Desert which is projected to be unsuitable (Fig. 1A). Most countries in South America as well as some countries in Asia (i.e., Thailand, Myanmar, Bangladesh, Indonesia, and Malaysia), showed high or optimal suitability for *P. absoluta* establishment (Fig. 1A). Globally, under the SSP2-4.5 scenario of the year 2050, the suitable areas under current conditions will remain favorable for the pest's establishment but an increase in suitability is expected in some parts of Africa (e.g. South Africa, Botswana, and Namibia) and the Southern part of Australia (Fig. 1B). Although the optimal areas will dramatically decrease across the globe under the SSP2-4.5 scenario of the year 2050, they will remain highly suitable for *P. absoluta* to thrive (Fig. 1C). The same trend is projected for the year 2070 under both SSP2-4.5 (Fig. 1D) and SSP5-8.5 (Fig. 1E) scenarios, where ERI will be changing from optimal to suitable or highly suitable in most parts of Africa, South America, and Asia.

**Figure 2:**
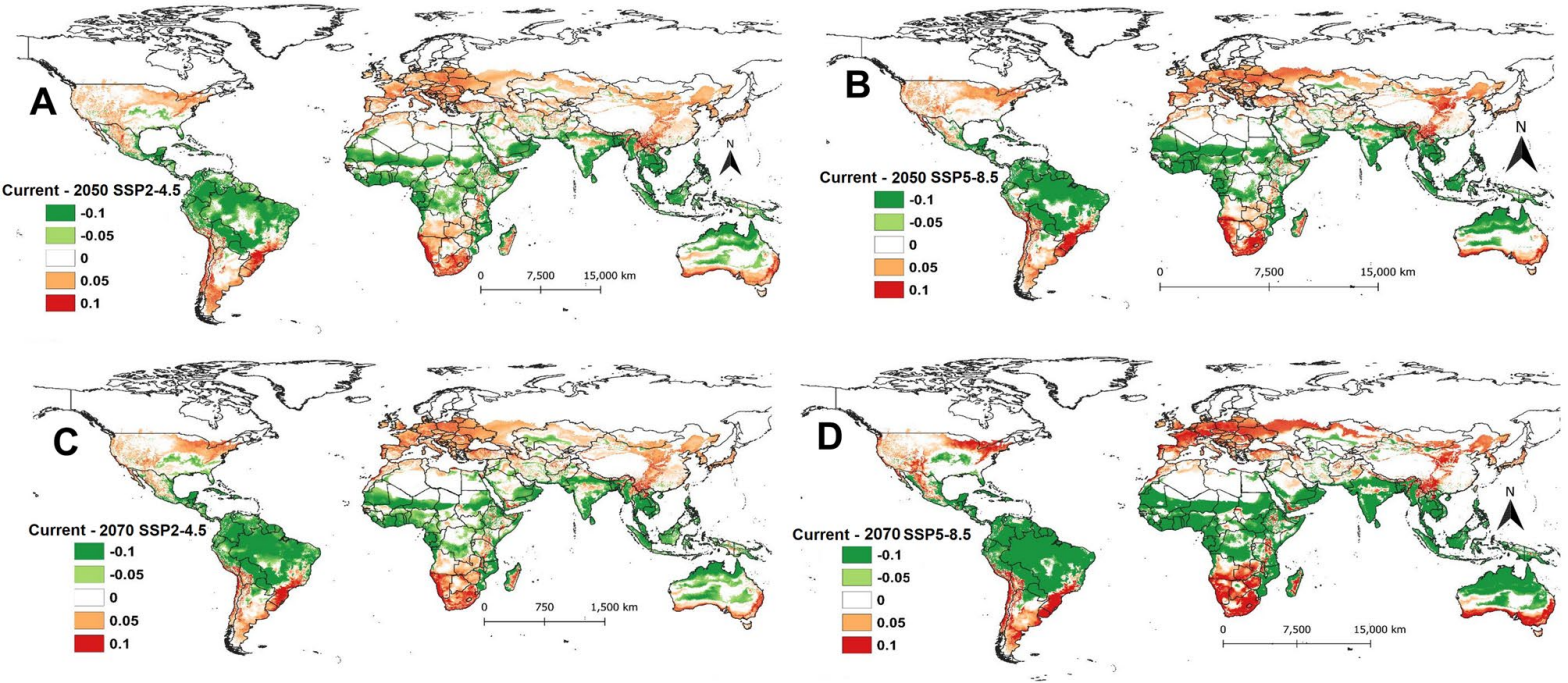
The change in establishment risk index (ERI) of *Phthorimaea absoluta* between current and future climatic scenarios for the years 2050 and 2070: (**A**) difference between current and future scenarios for 2050 (SSP2-4.5), (**B**) difference between current and future scenarios for 2050 (SSP5-8.5), (**C**) difference between current and future scenarios for 2070 (SSP2-4.5) and (**D**) difference between current and future scenarios for 2070 (SSP5-8.5).

In the future, a major decrease in ERI is expected in South America, Central and West Africa, Australia, and Asia (India, Thailand, Myanmar, Cambodia, Philippines, Bangladesh, Indonesia, and Malaysia) under different SSPs for the years 2050 and 2070 (Fig. 2). The ERI is expected to increase by 0.05 under the SSP2-4.5 scenario for the year 2050 in most parts of Europe, the USA, the Middle East, some parts of Mexico and Asian countries (Fig. 2A). ERI might also increase by 0.1 in South America (Chile, Argentina, and Southern Brazil), and Africa (South Africa, Botswana, some parts of Madagascar, Kenya, Tanzania, and Ethiopia) (Fig. 2A) by 2050 under the same scenario. A similar increase is also expected under the SSP5-8.5 scenario for the year 2050 (Fig. 2B) and the SSP2-4.5 scenario for the year 2070 (Fig. 2C) for these countries. However, the highest increase in ERI is projected for the SSP5-8.5 scenario for the year 2070, where the establishment risk of the pest will increase by 0.1 in most parts of Europe, the USA as well as in some African countries (South Africa, Botswana, Namibia, Angola, Zambia, and Zimbabwe) (Fig. 2D). Also, an increase in ERI by 0.1 is projected for year 2070 in some parts of Mongolia and China and southern Australia under the SSP5-8.5 scenario (Fig. 2D).

From the total area of 30001150.78 km^2^ of the African continent, 41.5% (12461040 km^2^), 25% (7500600 km^2^), and 18.3% (5504112 km^2^) are projected, respectively, to be suitable, highly suitable, and optimal for *P. absoluta* establishment under current climatic conditions (Table 1). Although the suitable areas are projected to decrease in 2050 by 4.7% (1412640 km^2^) and 7.9% (2364228 km^2^) under SSP2-4.5 and SSP5-8.5 scenarios, respectively, the highly suitable areas are projected to gain an additional 1121688 km^2^ (3.7%) and 10249416 km^2^ (9.2%) under the same scenarios, respectively (Table 1). A similar increase is also projected for the SSPs of the year 2070. In Asia, the marginally suitable areas (ERI = 0.2–0.4) will increase between 12.4 and 24%, while the suitable areas (ERI = 0.4–0.6) will increase by 0.2–1.3% depending on SSPs scenarios of the years 2050 and 2070 (Table 1). In Europe, unsuitable areas will be decreasing under all SSPs, and this will lead to an increase in the percentage of marginally suitable and suitable areas in the future. A similar trend is also expected in North America in the future, but the optimal areas are expected to decrease by 1.7% (404676 km^2^) and 2% (480816 km^2^) under SSP5-8.5 for the year 2050 and 2070, respectively, from the total area of the continent (24189364.53 km^2^) (Table 1). The optimal areas for *P. absoluta* establishment are expected to dramatically decrease in South America with a total area of 2108592 km^2^ (11.9%) and 5813856 km^2^ (32.7%) under SSP2-4.5 and SSP5-8.5 of the year 2050, respectively, and 6146928 km^2^ (34.6) and 7327908 km^2^ (41.3%) under SSP2-4.5 and SSP5-8.5 of the year 2070, respectively (Table 1).

**Table 1:** Projected area (km^2^) for *Phthorimaea absoluta* establishment risk index (ERI) and the percent of area changes between current and future climatic scenarios for the years 2050 and 2070 under two Shared Socio-economic Pathways (SSPs) (SSP2-4.5 and SSP5-8.5(SSPs).

Continent	ERI	Current		SSP2-4.5 /2050s		SSP5-8.5/2050s		SSP2-4.5 /2070s		SSP5-8.5/2070s	
		Area km^2^	% area of total	Area km^2^	% area of total	Area km^2^	% area of total	Area km^2^	% area of total	Area km^2^	%area of total
Africa	0.0–0.2	3368628	11.22	3656988	12.2 (0.9)*	3443472	11.48 (0.3)	3665412	12.21(0.9)	4396032	14.65 (3.4)
	0.2–0.4	1166724	3.89	1254852	4.18 (0.3)	2814912	9.38 (5.5)	3138912	10.46 (6.6)	3876660	12.92 (9.0)
	0.4–0.6	12461040	41.54	11048400	36.83 (− 4.7)	10096812	33.65 (− 7.9)	10678392	35.59 (− 5.9)	8841960	29.47 (− 12.1)
	0.6–0.8	7500600	25.00	8622288	28.74 (3.7)	10249416	34.16 (9.2)	8660844	28.87 (3.9)	10264968	34.21 (9.2)
	0.8–1.0	5504112	18.34	5418576	18.06 (− 0.3)	3396492	11.32 (− 7.0)	3857544	12.86 (− 5.5)	2621484	8.74 (− 9.6)
Asia	0.0–0.2	16330657	36.41	11569153	25.8 (− 10.6)	10023997	22.4 (− 14.1)	11416873	25.45(− 10.9)	7499713	16.72 (− 19.7)
	0.2–0.4	17119188	38.17	22676112	50.6 (12.4)	24423120	54.45 (16.3)	22892220	51.04 (12.9)	28072332	62.59 (24.4)
	0.4–0.6	5247180	11.69	5837508	13.02 (1.3)	5841072	13.02 (1.3)	5797008	12.92 (1.2)	5320080	11.86 (0.2)
	0.6–0.8	3508596	7.82	3382884	7.54 (− 0.3)	3371868	7.52 (− 0.3)	3367008	7.51 (− 0.3)	2992464	6.67 (− 1.2)
	0.8–1.0	2646108	5.89	1386072	3.09 (− 2.8)	1191672	2.66 (− 3.2)	1378620	3.07 (− 2.8)	967140	2.16 (− 3.7)
Europe	0.0–0.2	3837780	38.77	2238372	22.6(− 16.2)	602172.925	6.08 (− 32.7)	1825777	18.4 (− 20.3)	871956.9	8.81(− 29.9)
	0.2–0.4	5912424	59.73	7327152	74.0 (14.3)	9000936	90.93 (31.2)	7825104	79.05 (19.3)	8805132	88.95 (29.2)
	0.4–0.6	148392	1.49	332100	3.36 (1.9)	294192	2.97 (1.48)	246708	2.49 (1)	219744	2.22 (0.7)
	0.6–0.8	0	0	972	0.01 (0.01)	1296	0.013 (0.01)	1008	0.01 (0.01)	1764	0.017 (0.02)
North America	0.0–0.2	12698704	52.49	10430380	43.12 (− 9.4)	9395200.53	38.8 (− 13.7)	9798581	40.51(− 11.9)	6818105	28.18 (− 24.3)
	0.2–0.4	7960356	32.91	10402344	43.0 (10.1)	11370456	47.01 (14.1)	11120976	45.97 (13.1)	14185368	58.64 (25.7)
	0.4–0.6	1947564	8.05	2045412	8.46 (0.4)	2206764	9.12 (1.07)	2039580	8.43 (0.4)	2279988	9.43 (1.4)
	0.6–0.8	1002132	4.14	1077948	4.46 (0.3)	1041012	4.30 (0.2)	1040040	4.29 (0.2)	806112	3.33 (− 0.8)
	0.8–1.0	580608	2.40	233280	0.96 (− 1.4)	175932	0.73 (− 1.7)	190188	0.79 (− 1.6)	99792	0.41 (− 1.99)
South America	0.0–0.2	394854	2.22	167406	0.94 (− 1.3)	217626	1.23 (− 0.9)	281454	1.58 (− 0.6)	169350.9	0.95 (− 1.3)
	0.2–0.4	2048328	11.53	1895724	10.68 (− 0.9)	1789452	10.08 (− 1.5)	1721088	9.69 (− 1.8)	1569456	8.84 (− 2.7)
	0.4–0.6	2308500	13.00	3383532	19.05 (6.1)	3879900	21.85 (8.9)	3759696	21.17 (8.2)	5913648	33.30 (20.3)
	0.6–0.8	4482216	25.24	5895828	33.20 (7.9)	9160776	51.59 (26.4)	9618588	54.17 (28.9)	8909352	50.17 (24.9)
	0.8–1.0	8523792	48.00	6415200	36.1 (− 11.9)	2709936	15.3 (− 32.7)	2376864	13.4 (− 34.6)	1195884	6.73 (− 41.3)
Australia	0.0–0.2	504243	6.55	1401903	18.2 (11.7)	1399131.08	18.17 (11.6)	1400643	18.19 (11.6)	1399635	18.17 (11.6)
	0.2–0.4	507456	6.59	288036	3.74 (− 2.9)	1020852	13.31 (6.9)	510552	6.63 (0.04)	1757448	22.82 (16.2)
	0.4–0.6	5891616	76.49	5621616	72.99 (− 3.5)	4903920	63.67 (12.8)	5381208	69.87 (− 6.6)	4178412	54.25 (− 22.2)
	0.6–0.8	774432	10.06	386820	5.02 (− 5.0)	375228	4.87 (− 5.2)	402444	5.23 (− 4.8)	365400	4.74 (− 5.3)
	0.8–1.0	23904	0.31	3276	0.04 (− 0.3)	2520	0.03 (− 0.3)	6804	0.09 (− 0.2)	756	0.01 (− 0.3)

The asterisk represents the change in areas (gain or loss) between current and future climate scenarios.

### Generation index of *Phthorimaea absoluta*

**Figure 3:**
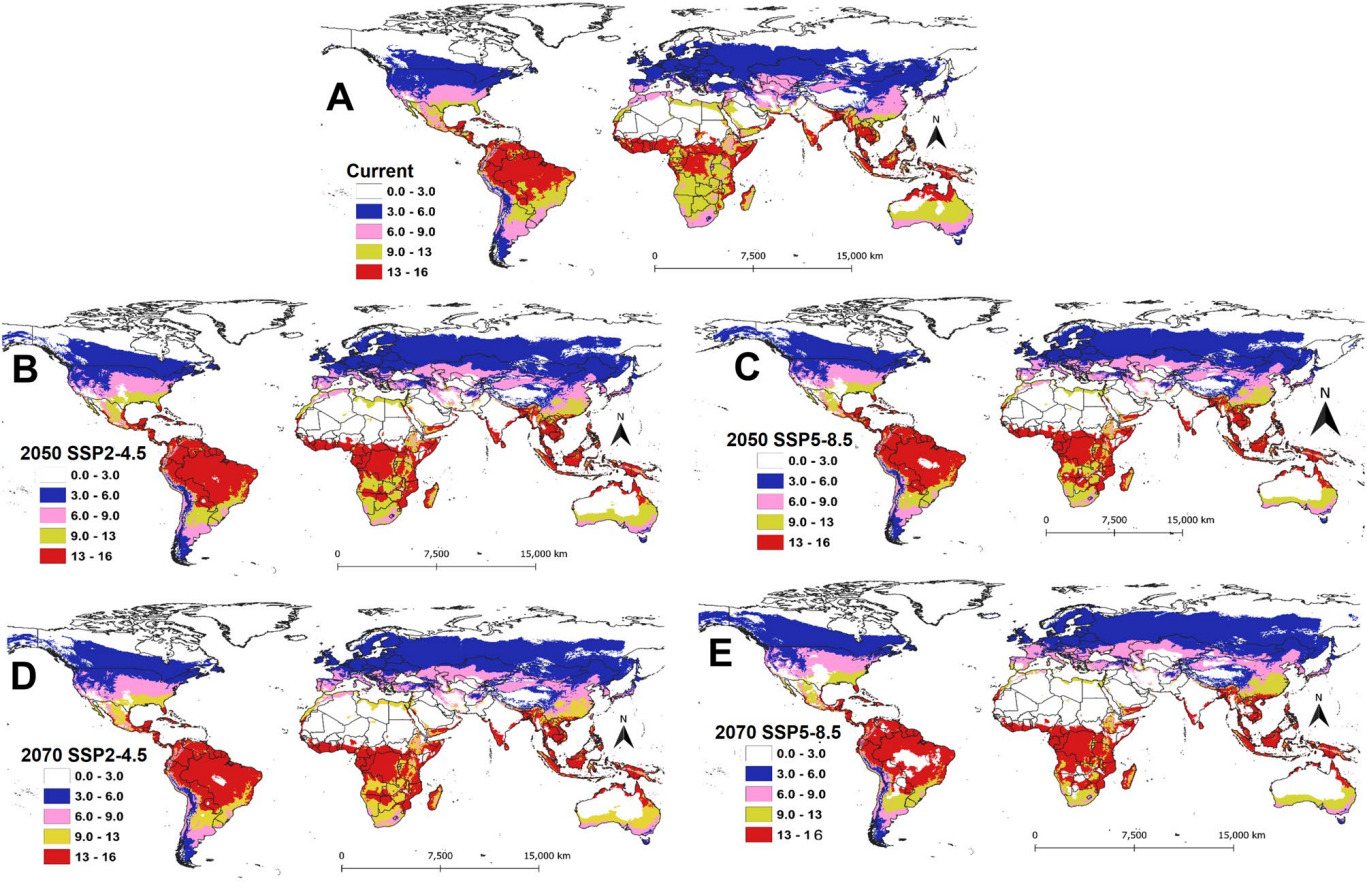
Generation Index (GI) of *Phthorimaea absoluta* projected worldwide using temperature-based phenology model under (**A**) current climatic condition, (**B**) future scenarios for 2050 (SSP2-4.5), (**C**) future scenarios for 2050 (SSP5-8.5), (**D**) future scenarios for 2070 (SSP2-4.5) and (**E**) future scenarios for 2070 (SSP5-8.5).

**Figure 4:**
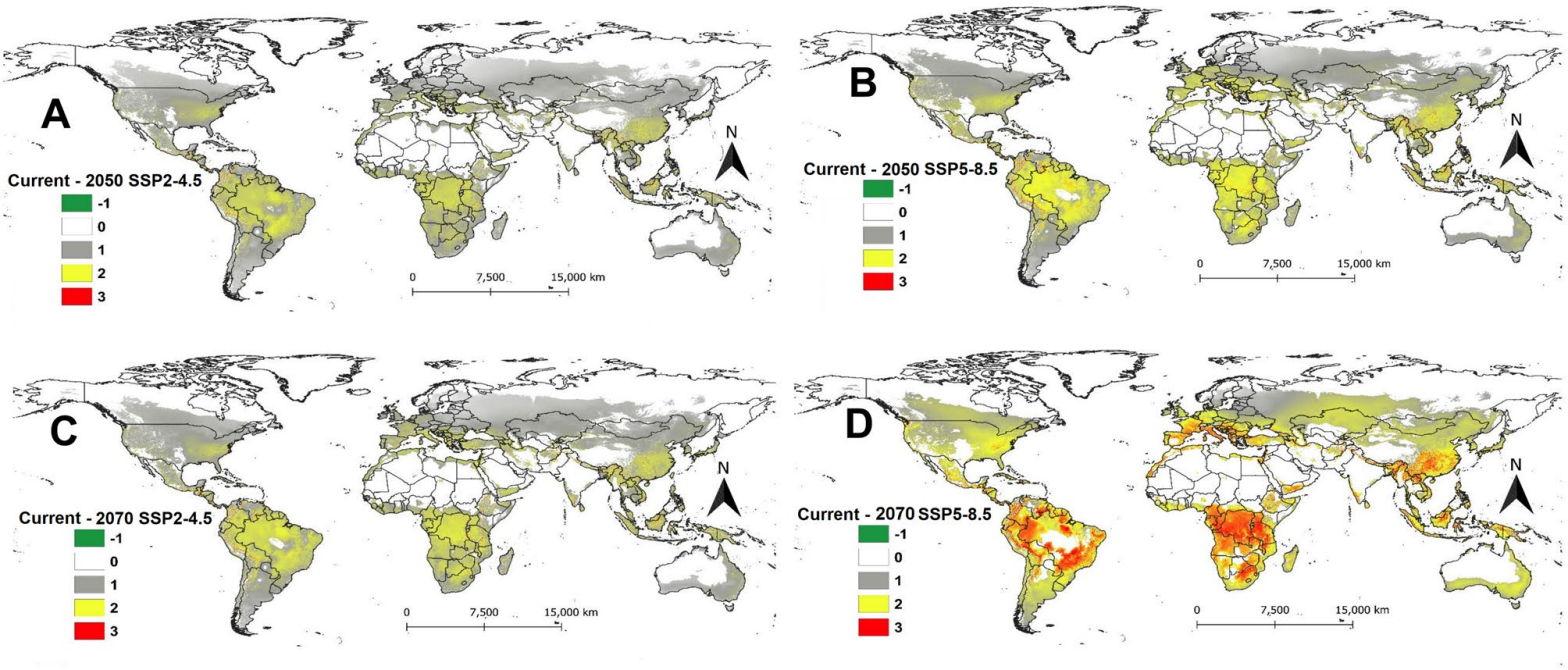
The change in generation index (GI) of *Phthorimaea absoluta* between current and future climatic scenarios for the years 2050 and 2070: (**A**) difference between current and future scenarios for 2050 (SSP2-4.5), (**B**) difference between current and future scenarios of 2050 (SSP5-8.5), (**C**) difference between current and future scenarios for 2070 (SSP2-4.5) and (**D**) difference between current and future scenarios for 2070 (SSP5-8.5).

The generation index, which computes the mean number of generations that *P. absoluta* could complete per year under different climatic scenarios, is shown in Fig. 3. According to the model projection, in South America and sub-Saharan Africa, *P. absoluta* can complete between 6 and 16 generations per year under the current climatic scenario depending on the location. This situation is also expected in future SSPs but with an increased number of generations in some areas for the two regions (Figs. 3 and 4). However, in most European countries, *P. absoluta* can only complete between 3 and 6 generations per year except for Spain, Portugal, and Italy, where the pest can complete between 6 and 9 generations per year (Fig. 3A). Under SSPs of the year 2050, the number of generations per year is expected to increase across Europe, Asia, and the USA (Fig. 3A, B). A similar trend is projected under SSP2-4.5 (Fig. 3C) and SSP5-8.5 (Fig. 3D) scenarios for the year 2070.

The change in the number of generations for *P. absoluta* between the current and future climatic scenarios is shown in Fig. 4. Due to the expected increase in temperature, an additional one generation per year is predicted in most parts of the world especially Europe, Asia, and North America in the year 2050 under the SSP2-4.5 scenario (Fig. 4A). For the same year, under the SSP5-8.5 scenario, the number of generations of *P. absoluta* is expected to increase by two more generations per year across Europe, Brazil, and sub-Saharan Africa, especially the Democratic Republic of Congo, Tanzania, Congo, Gabon, Zambia, Malawi, Angola, Namibia, Botswana, South Africa and Cameroon (Fig. 4B). Globally, in the year 2070, the trend of the generation index under the SSP2-4.5 scenario is expected to be similar to that for SSP2-4.5 for the year 2050, except that the pest could produce an additional two generations across Brazil and the Democratic Republic of Congo (Fig. 4C). Similarly, under SSP5-8.5 for the year 2070, *P. absoluta* generation could increase by two to three generations per year, especially in Europe, sub-Saharan Africa, and some parts of the USA and China (Fig. 4D).

### Activity index of *Phthorimaea absoluta*

**Figure 5:**
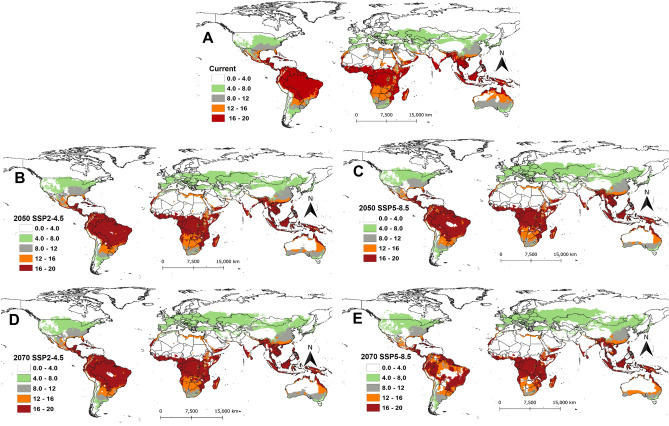
Activity index (AI) of *Phthorimaea absoluta* projected worldwide using temperature-based phenology model under (**A**) current climatic condition, (**B**) future scenarios for 2050 (SSP2-4.5), (**C**) future scenarios for 2050 (SSP5-8.5), (**D**) future scenarios for 2070 (SSP2-4.5) and (**E**) future scenarios for 2070 (SSP5-8.5).

The activity index (AI) which indicates the potential population growth rate of *P. absoluta* throughout the year under current and future climatic scenarios is shown in Fig. 5. Under the current climatic scenario, the potential activity index (AI) of *P. absoluta* is projected to be high in the tropical and subtropical regions, regardless of the climatic scenario, compared to the temperate regions, such as Europe, the USA, Mongolia, and China (Fig. 5A).

In the year 2050, the AI will remain high in Brazil, Mexico, Venezuela, Colombia, Ecuador, Peru, Bolivia, and Paraguay under both SSP2-4.5 (Fig. 5B) and SSP5-8.5 (Fig. 5C). The same is true for sub-Saharan African countries where AI will remain high under future climatic conditions (Fig. 5B, C). The potential AI of *P. absoluta* under SSP2-4.5 for the year 2070 is expected to be similar to that of the year 2050 with high AI projected in India, Thailand, Myanmar, Vietnam, Cambodia, Philippines, Bangladesh, Indonesia, and Malaysia (Fig. 5D). However, under SSP5-8.5 for the year 2070, the AI might decrease in Brazil and some parts of Bolivia, Paraguay, and Argentina (Fig. 5E).

**Figure 6:**
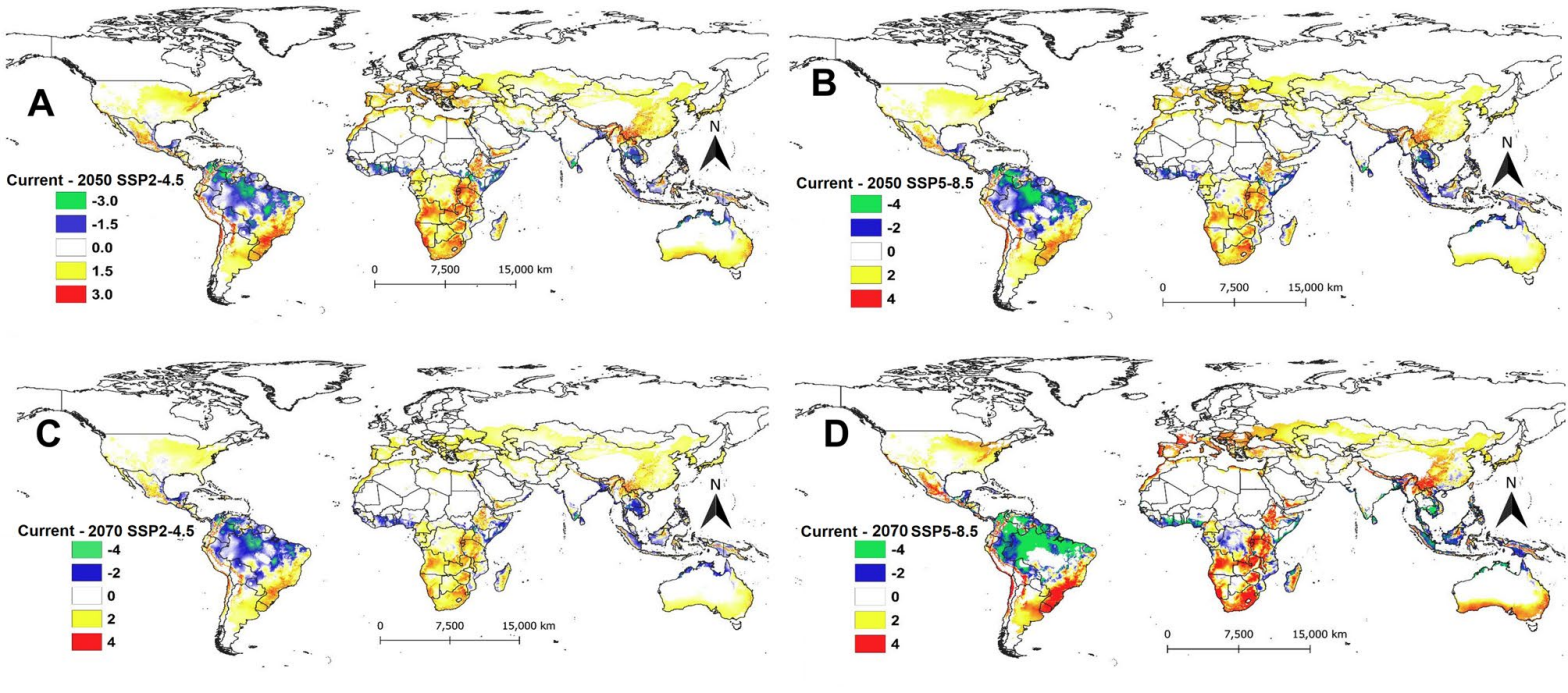
The change in generation index (GI) of *Phthorimaea absoluta* between current and future climatic scenarios for the years 2050 and 2070: (**A**) difference between current and future scenarios fir 2050 (SSP2-4.5), (**B**) difference between current and future scenarios for 2050 (SSP5-8.5), (**C**) difference between current and future scenarios for 2070 (SSP2-4.5) and (**D**) difference between current and future scenarios for 2070 (SSP5-8.5).

The change in AI between current and future climatic scenarios is shown in Fig. 6. An increase in AI by 1 indicates that the population will increase by ten-fold over the current scenario. Overall, the AI is expected to increase between 1 and 4, indicating an increase in *P. absoluta* population by 10–40-fold per year (Fig. 6). An increase in AI by 1 to 3 is expected in 2050 under both SSP2-4.5 (Fig. 6A) and SSP5-8.5 (Fig. 6B) in most sub-Saharan African countries, except Somalia, Nigeria, Benin, Ghana, and Ivory Coast, where AI is expected to decrease by 1. In South America, a decrease in AI between 2 and 4 is projected for Brazil, Venezuela, Colombia, Ecuador, Peru, and Bolivia under both SSPs of 2050 (Fig. 6A, B) and SSP2-4.5 for the year 2070 (Fig. 6C). Most parts of Asia also showed an increase in AI by 2 under the same scenarios with the exception of Thailand, Myanmar, Vietnam, Cambodia, Philippines, Bangladesh, Indonesia, and Malaysia where AI might decrease by 2 (Fig. 6A, B). The highest increase in AI is projected under SSP5-8.5 for the year 2070 in most parts of Europe, Asia, and East and South Africa (Fig. 6D).

## Discussion

In this study, we used a complete life cycle of *P. absoluta* (phenology model) described by Mohamed et al.^31^ to predict its establishment and number of generations under current and future climatic scenarios using insect life cycle modelling software. The phenology model calculated the thermal requirement for each developmental parameter including the development time, development rate, mortality rate, fecundity, adult senescence, and the life table parameters that determine the population growth rate. The advantage of this method is that it provides comprehensive information on the effect of temperature on the overall insect growth, and therefore a more accurate prediction of its potential geographical distribution under different climate change scenarios. Our predictions indicate worsening scenarios of the impact of *P. absoluta* in Europe, Asia, and South and East Africa under future climatic conditions in terms of establishment, increased number of generations and activity indices, which might lead to more potential damage to tomato and other host crops.

Under current climate conditions, our results showed that *P. absoluta* could permanently establish itself in tropical and sub-tropical regions (ERI ≥ 0.6). Our model prediction closely matches the current distribution of this pest worldwide. For example, *P. absoluta* has invaded most parts of sub-Saharan Africa, where our model showed ERI between 0.4 and 1. Similarly, some parts of Spain, the country where *P. absoluta* was first detected following its transatlantic invasion^4,5^, showed high suitability for its establishment (ERI = 0.4–0.6). Also, the outcome of our model prediction for the current scenario was similar to that of Santana et al.^29^. Using a CLIMEX model, the authors projected large suitable areas for *P. absoluta* establishment in Central and South Americas, Africa, Asia and Australia under the current climatic scenario, where our model also showed high climate suitability for this pest to thrive. Likewise, Desneux et al.^4^ projected some parts of Tunisia, Algeria and Morocco in North Africa to be suitable for *T. absoluta*, which agrees with our findings that showed suitable climate conditions for this pest to establish in these areas under the current climate scenario.

Although our model showed high climate suitability for *P. absoluta*, in the Central African Republic, Congo, Gabon, Ivory Coast, Liberia, and Sierra Leone, as well as some Asian countries, the pest is yet to be officially reported in these countries^32^. Nonetheless, *P. absoluta* could spread into suitable uninvaded areas as it was recently reported in Togo^6^, where the model predicted high climate suitability. Contrary to the finding by Tonnang et al.^27^ who predicted a high risk of the establishment of the pest across the USA and some parts of Canada under the current situation, our model projected marginal suitability for *P. absoluta* establishment in these countries except of some parts in Southern USA. The discrepancy between the outcome of the two studies could be explained by the different datasets that were used for prediction. Tonnang et al.^27^ used records for *P. absoluta* occurrence in South America to predict the potential distribution of the pest worldwide. In addition, the authors used thermal requirements for *P. absoluta* obtained from a degree day model, unlike our study which used a complete phenology model that described the thermal requirements of the complete life cycle of the pest. In fact, the degree day model predicts only the development rate of insect pest, and the use of such model has some limitations. For example, the development rate of insect increases linearly with an increase in temperature until the optimum point, and then, it decreases until reaching the highest thermal threshold for development. However, such an increase in the development rate does not always translate to an improvement in the overall performance for the insect. The optimal temperature for development rate usually leads to high mortality of immature stages, and subsequently lower population growth rate^33^. Unlike the degree day model, the phenology model provides the thermal requirements for a complete life cycle of the pest including development time, development rate, mortality rate, fecundity, adult senescence, and the life table parameters that determine the population growth rate. We considered all these parameters in our study to predict the suitable areas for *P. absoluta* establishment.

Based on our model prediction, under future climatic scenarios (the years 2050 and 2070), the ERI will increase across Europe, North America, Northern Asia, South Australia, and South and East Africa. However, the establishment risk for *P. absoluta* is expected to be higher under SSP5-8.5 scenario compared to SSP2-4.5. Although our results under current conditions were in line with those reported by Santana et al.^29^, the projected future scenarios were slightly different. For example, Santana et al.^29^, projected West and Central Africa to be unsuitable for *P. absoluta* under SRES A2 scenario for the year 2050 (= SSP5-8.5), while our results showed a high risk of establishment in these areas. Indeed, *P. absoluta* has already invaded many countries in West and Central Africa where the pest is currently causing huge damage to small-scale farmers^32^. Similarly, our future projection for India also differed from that of Fand et al.^30^ who projected marginal habitat suitability for *P. absoluta* occurrence using the Maxent model, compared to our model that showed moderate to high suitability for *P. absoluta* establishment. These discrepancies may be explained by the different climatic datasets used for modelling as Santana et al.^29^ used SRES A2 scenario and Fand et al.^30^ used A1B emission scenario, compared to SSPs scenarios that we used. In fact, the reliability of future climate projections has some level of uncertainty^34^, thus different climate models may produce different results when used in species distribution modelling. The second explanation for these differences could be attributed to different modelling tools that were used by these studies which have different levels of complexity in their fitting.

Compared to the tropical regions, the projected ERI for *P. absoluta* in the temperate region is relatively low (ERI = 0.2–0.4), although the pest is present in part of this region causing severe damage to tomato crops^27,35^. This is mainly attributed to the extreme temperature variation within the year in temperate regions which limits the survival of immature stages for a few months. Our prediction indicates that all life stages of *P. absoluta* could survive between 3 and 5 months per year in Europe and other temperate regions. In this region, the lower temperature reaches below zero degree Celsius in winter, which is not conducive for the survival of *P. absoluta*. However, some studies revealed that the pest goes into diapause during winter and successfully passes the harsh environmental conditions^8–10,36^, and this explains the occurrence of the pest in temperate regions.

The present study showed that *P. absoluta* could complete 3–16 generations per year, depending on the environmental conditions. A study by Abolmaaty et al.^37^ predicted 11–13 generations per year under open field conditions in Egypt using a degree-day accumulation model. This agreed with our results that showed *P. absoluta* can produce between 9 and 13 generations per year in Egypt under current conditions. Martins et al.^38^ reported 17 generations per year at a constant temperature of 33 °C, while de Campos et al.^9^ argued that the pest could complete between 6 and 9 generations per year in Mediterranean Basin under a warmer temperate climate, which also is in line with our findings. Our model also predicted an increase in the number of generations per year in many locations under future climatic conditions. For example, an additional 2–3 generations per year are expected by the years 2050 and 2070 in most parts of Europe, Africa, Asia, and South America. This is mainly due to the expected temperature rise, as a result of global warming, which will lead to a shorter developmental time of *P. absoluta*, hence more generations per year.

The AI, which is computed based on the finite rate of population increase (λ), served as a good indicator of the potential population growth rate and it signifies the spread risk and severity of the insect pest. Under current conditions, AI ranged between 2 and 20 in areas where *P. absoluta* could well establish and cause damage. This was similar to AI of closely related species *Phthorimaea operculella* in potato production areas worldwide which ranged between 1 and 24^17^. However, under future scenarios, this index will increase by 2–4 in most parts of sub-Saharan Africa, Asia, and Europe. This implies that the populations of *P. absoluta* will increase by 20–40-fold per year in these areas under future conditions if the temperature is considered to be the only factor that affects the pest population. As the number of generations for *P. absoluta* increases, the population abundance and damage (AI) for the pest is also increasing. Thus, the increase in the number of generations within the cropping season or year as a result of climate change may aggravate the risk of *P. absoluta* under future climatic conditions, thus causing more damage to the crops.

The change in temperature significantly affects the distribution, abundance, reproductive success and establishment of the insect pest^18,20^. Therefore, the increase in temperature as a result of global warming may have a serious effect on the potential distribution and spread of *P. absoluta* toward new areas and this might lead to huge crop losses. In this regard, our study could serve as an early warning tool that could guide policy makers to prevent the future spread of *P. absoluta* into new areas and curb the current invasion through the development of effective phytosanitary measures and efficient control strategies. Identification of suitable regions that are at high risk of invasion by *P. absoluta* is very important for pest surveillance and monitoring. Therefore, our results will be useful for regulatory bodies such as national plant protection, particularly in uninvaded countries to determine pest risk analysis areas to prevent the invasion of *P. absoluta* and safeguard future spread.

### Model limitations

In this study, we provided predictions that used a combination of temperature data and *P. absoluta* physiological parameters. Our model accurately predicted the current distribution of the pest as the real-world records of *P. absoluta* distribution closely aligned with our findings. However, one of the limitations of the current study is that the results obtained are based only on temperature as a factor influencing the distribution of *P. absoluta* and it does not consider other factors such as host plant, natural enemies, rainfall, and relative humidity. Also, the model does not consider the areas in which the climatic conditions could be artificially changed such as the use of greenhouses. This is very important for countries such as North America, where about 20% of the crop is produced in greenhouses^39^. An important observation is a discrepancy between models’ outputs. In this regard, we propose an ensemble approach that combines features and skills obtained from the individual modeling methods. Therefore, a “generalizer” meta-mapping that learns from a weighted combination of the key intrinsic features embedded in each suitability map could be applied to improve accuracy relative to any individual map obtained from a single model.

## Materials and methods

### Modelling tool

In this study, we used insect life cycle modelling (ILCYM version 3.0) tool^17,20^ to predict the future distribution and number of generations of *P. absoluta* under different climatic scenarios. ILCYM consists of three modules, namely the model builder module, the validation and simulations module, and the population analysis and mapping module that is used to study the population ecology of insect pests^20^. The model builder contains several empirical linear and nonlinear functions that are used to predict the effect of temperature on insect developmental time, developmental rate, mortality rate, oviposition and adult longevity based on life table data collected at different constant temperatures. These functions are known as phenology models or process-based models, and they are used to determine the relationship between temperature and insect development. The validation and simulations module in ILCYM compiles the all the function in one phenology model that is used to estimate the life table parameters that determine the population growth rate of insect pests under both constant and fluctuating temperatures. This module also validates the phenology model using life table data of the insect pests collected under fluctuating temperatures. Finally, the population analysis and mapping module links the phenology model with geographical information system (GIS) platforms that allow spatial simulations and estimation of the pest risk indices based on insect life cycle and temperature data^17,20^. In our previous study by Mohamed et al.^31^, we used model builder and validation and simulations modules to construct, simulate and validate the temperature-dependent phenology models of *P. absoluta*. In the current study, we complied these models and used them in the population analysis and mapping module in ILCYM to spatially predict three risk indices of *P. absoluta* including establishment risk index (ERI), generation index (GI), and activity index (AI) of *P. absoluta*.

### Temperature-dependent phenology model for *Phthorimaea absoluta*

**Figure 7:**
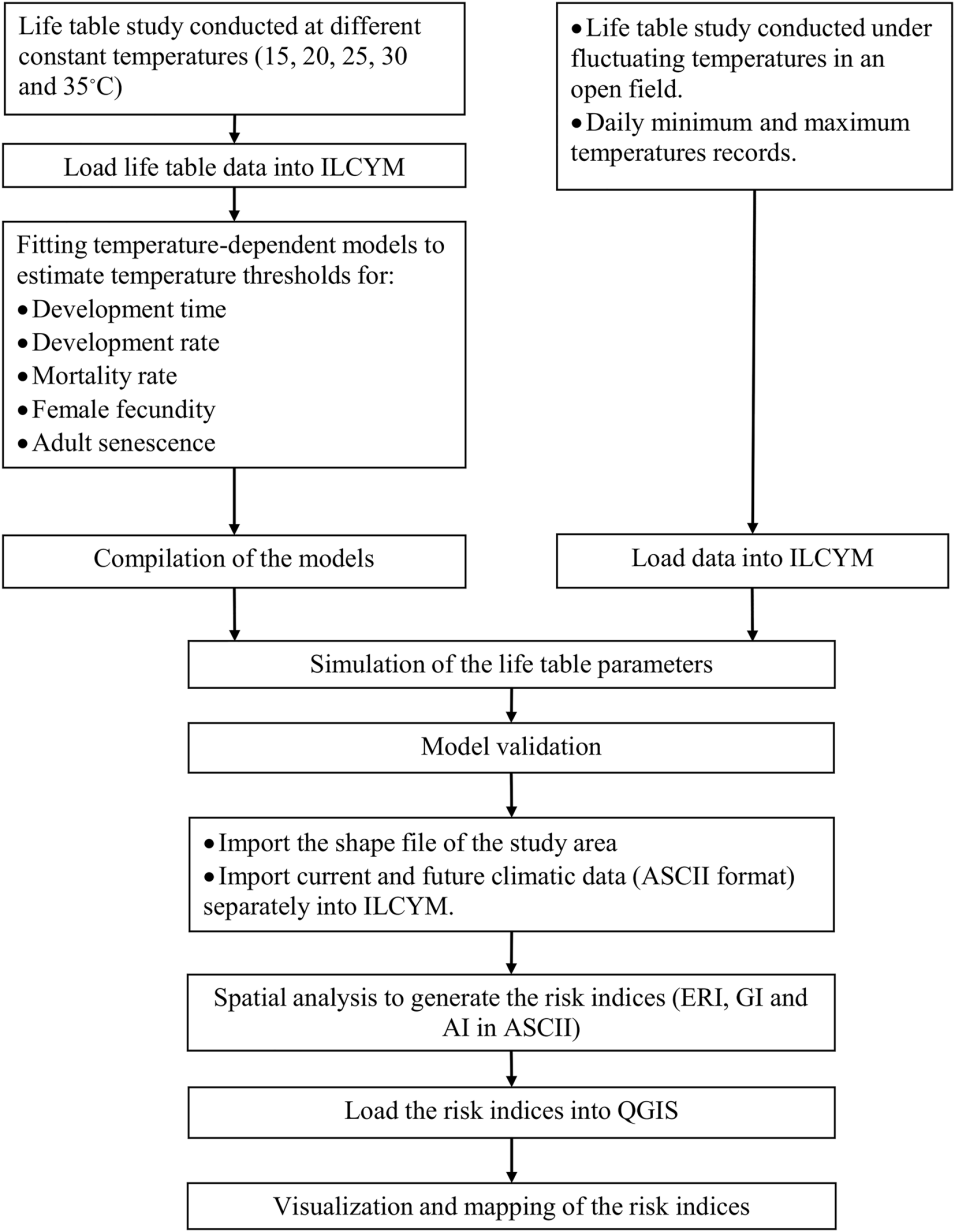
The scheme implemented in Insect Life Cycle Modelling (ILCYM) and QGIS software to simulate and map the risk indices of *Phthorimaea absoluta*.

A temperature-dependent phenology model for *P. absoluta* developed by Mohamed et al.^31^ was used to predict three risk indices of the pest viz., ERIGI, and AI, under current and future climatic conditions. The phenology model was developed in ILCYM (version 3)^20^ using life table data of *P. absoluta* collected in the laboratory at five constants temperatures of 15, 20, 25, 30, and 35 °C (Fig. 7). The developed model consists of a set of mathematical functions that were used to describe temperature dependency of development time, development rate, and mortality rate of *P. absoluta* immature stages as well as female fecundity and adult senescence (see Mohamed et al.^31^ for more details about the functions). These functions include the complementary log–log (CLL) fitted to the normalized development time of egg and pupal stages as well as female and male longevity; logit function fitted to the normalized development time of larval stage; Sharpe and DeMichele function 1^40^ fitted to the development rate of egg and larva; Briere function 1^41^ fitted to the development rate of pupa, Wang function 1^42^ fitted to the mortality rate of all immature stages; Exponential modified function 3 fitted to cumulative fecundity; Taylor function 1 fitted to mean fecundity of the female, and Exponential simple function fitted the male and female senescence. These functions were compiled (phenology model) and validated with life table data collected from a study conducted under fluctuating temperatures (Fig. 7) (see Mohamed et al.^31^ for more details). The model predicted that a temperature range between 20 and 25 °C is optimal for *P. absoluta* immature stages development in which high population growth is occurring with an intrinsic rate of increase ranging between 0.09 and 0.13^31^.

### Temperature data

Monthly minimum and maximum temperature data (2.5 arcmin) were obtained from WorldClim database (http://www.worldclim.org/) and used to estimate the risk indices of *P. absoluta* under current and future climatic scenarios. To simulate the risk under the current scenario, we used interpolated historical temperature data of the years 2015. For future climatic scenarios, data of the Beijing Climate Center Climate System Model (BCC-CSM)^43^ for the years 2050 and 2070 under two Shared Socio-economic Pathways (SSPs) SSP2-4.5, and SSP5-8.5 were used to simulate the impact of temperature increase on the invasion risk of *P. absoluta*. Data were downloaded in “TIF” format and then were converted to “ASCII” format before they were used for the spatial analysis.

### Spatial analysis and mapping of the risk indices

The scheme implemented to simulate *P. absoluta* risk indices is shown in Fig. 7. The developed temperature-dependent phenology model was used to simulate the life table parameters of *P. absoluta*. These parameters include the gross and net reproductive rates, mean generation time, intrinsic rate of population increase, doubling time, and the finite rate of population increase as reported in Mohamed et al.^31^. The simulations considered the daily minimum and maximum temperatures as a cosine function to calculate temperature-dependent population parameters at each 15 min time step while accounting for daily temperature variability as described by Kroschel et al.^17^. The mathematical equation developed by these authors was used to estimate the temperature of the first half-day

$${T}_{i}=\left(\frac{Max-Min}{2}\right)\times cos\left(\frac{\pi \times (i-0.5}{48}\right)+\left(\frac{Max+Min}{2}\right)$$

where ${T}_{i}$ is the temperature (°C) at time step $i$ ($i$ = 1, 2, 3, …, 48), and $Min$ and $Max$ are the daily minimum and maximum temperatures, respectively. The same equation was applied to calculate ${T}_{i}$ for the second half-day using the minimum temperature of the next day. We used average monthly temperature data obtained from WorldClim database. Hence the daily temperatures were replaced with monthly averages for the half-day temperature to predict each 15 min time step in the above function. Using the simulated population growth parameters of *P. absoluta* based on the phenology model and temperature data, the following risk indices were estimated under SSP2-4.5 and SSP5-8 5 of the years 2050 and 2070 and visualized using QGIS:

1. Establishment risk index (ERI).

The ERI identifies the suitable areas for pest establishment under a specific thermal condition and it is calculated based on the daily survival of the immature stage^17^ and the species reproduction ability^44^. ERI has probability values between 0 and 1, with 1 indicating a possibility for individuals in each immature life stage (egg, larva, pupa) to survive, reaching the adult stage (throughout the year; 365 days), and the adult laying eggs for the species life cycle to continue. The function used by Ngowi et al.^44^ was employed to compute ERI as follows:

$$ERI = \frac{{\mathop \sum \nolimits_{1}^{i = 365} I_{i} }}{{I_{I} }}*net - reproduction$$

where $ERI$ is the establishment risk index, ${I}_{i}$ is the number of days $i$ within the year ($i$ = 1, 2, 3, …, 365) and ${I}_{I}$ is the total number of days within the year (365 days).

(2) Generation index (GI).

Generation index herein refers to the number of generations that *P. absoluta* may produce in a specific location in one year. GI was calculated based on the mean generation time (in days) using the equation described by Kroschel et al.^17^ as follows:

$$GI = \frac{{\mathop \sum \nolimits_{i = 1}^{365} 365/Tc}}{365}$$

where $GI$ is the generation index (number of generations per year), 365 is the number of days in the year and $Tc$ is generation time (in days) calculated for each day $i$ ($i$ =1, 2, 3, …, 365).

(3) Activity index (IA).

The IA is strongly associated with the finite rate of population increase, and it takes into consideration the whole life history traits of the pest. AI was calculated by taking the log of the products of estimated finite rates of population increase calculated for each day of the year using function described by Kroschel et al.^17^ as follows:

$$AI = log_{{10}} \prod\limits_{{i = 1}}^{{365}} {\lambda _{i} }$$

where $AI$ is the activity index, ${\lambda }_{i}$ is the finite rate of the population increase within the year at each day $i$ ($i$ =1,2,3, …, 365). For instance, a value of 4 for this index indicates a potential population increase by a factor of 10,000 within a year.

## Data Availability

All data are available within the manuscript.
